# Enhancement of bioactivity and molecular docking analysis of bioglass loaded zein and sodium alginate composite beads for biomedical applications

**DOI:** 10.1038/s41598-025-95691-7

**Published:** 2025-04-18

**Authors:** A. A. Desoky, Khairy T. Ereiba, Ahmed M. Bakr, A. S. Abdraboh

**Affiliations:** 1 Physics Department, Faculty of Science, Al-Azhar University, Nasr City, Cairo 11884 Egypt; 2 Spectroscopy Department, Physics Research Division, National Research Centre, 33 El Bohouth St, Dokki, Giza 12622 Egypt

**Keywords:** Zein/SA, Beads, Bioglass, Bioactivity, Molecular Docking, Hydroxyapatite, Biophysics, Computational biology and bioinformatics, Molecular biology, Materials science

## Abstract

This study intends to conduct both in-vitro and in-silico investigations to assess the bioactivity of newly developed zein/sodium alginate (SA) composite beads. These beads, prepared using the dropwise method, were infused with different concentrations (10%, 20%, 30%, and 40%) of bioglass (BG). These BG-loaded zein/SA- composites were formed into beads with calcium chloride (CaCl_2_, 5%) as a crosslinking agent. The resulting composite beads were characterized using various techniques (XRD, FTIR, SEM, and EDXA) both before and after in vitro testing in Simulated Body Fluid (SBF). A 3D model of zein was constructed using I-TASSER and validated through PROCHECK and ERRAT servers. The interaction forces between the zein protein and sodium alginate were analyzed in silico using molecular docking with Autodock vina and PyMOL software. The results showed that the formation of a hydroxyapatite (HA) layer on the surface of the BG-loaded zein/SA composite beads confirmed their biological activity, which increased with higher BG content. The results suggest that zein/SA composite beads loaded with bioglass (BG), exhibiting good bioactivity, are suitable for use in bio-tissue engineering applications. A molecular docking study revealed that sodium alginate (SA) can interact with zein through van der Waals forces and hydrogen bonds, leading to the formation of stable complexes. This zein/SA complex is well-suited for carrying bioglass (BG) and has potential applications as a drug carrier in a drug delivery system. Based on the results, our study shows that BG-loaded Zein/SA composite beads are bioactive materials, and can be used in tissue engineering and improve the deposition of new HA, consequently enhancing bone generation. In addition, the prepared composite beads can serve as an excellent drug carrier (future work).

## Introduction

Biopolymers like zein can be combined with various bio-ceramics, such as bioactive glasses (BGs), hydroxyapatite, and types of calcium phosphate cement, to enhance the bone-binding capability of composites^1,2^. Bioactive glass (BG) is a popular bio-ceramic known for forming strong bonds with natural bone^3^. However, BG has poor mechanical properties under load and an elastic modulus higher than that of cortical bone, which can lead to stress shielding issues^4^. To address these limitations, BG is often integrated with biocompatible polymers^5^. Zein, a natural protein, is recognized for enhancing osteoblast cell proliferation and attachment^6,7^. Additionally, zein films have been shown to resist biofilm formation, which is crucial since biofilms and bacterial colonization pose significant health risks^8^. Therefore, the combination of zein and BG can offer strong bone-binding capability, resistance to biofilm formation, and improved osteoblast cell attachment and proliferation^9,10^. Zein, a type of prolamin protein, is made up of different polypeptide fractions that are classified into four categories: α, β, γ, and δ. The α-zein bands occur at 19 and 22 kDa, β-zein at 14 kDa, γ-zein at 16 and 27 kDa, and δ-zein at 10 kDa. Among these, α-zein is the most prevalent (70–85%), and γ-zein (10–20%), with both β-zein and δ-zein comprising 1–5% each. All zein fractions are amphiphilic, containing both hydrophobic and hydrophilic amino acids. α-zein is notable for its highly similar repeating units and high α-helix content^11^. Alginate is an anionic polysaccharide derived from sea algae, which carries a negative charge. It consists of linear block copolymers of 1–4 linked β-D-mannuronic acid (M) and α-L-guluronic acid (G) and offers benefits such as high biocompatibility, abundant availability, good hydrophilicity, and easy formability^12^. Previous research primarily focused on studying or evaluating the combination of BG and zein protein composites in the form of scaffolds or biofilms. PCL/zein composite coating was created to improve the biological activity of BG scaffolds by promoting faster hydroxyapatite (HA) formation. It was discovered that the inclusion of zein increases the degradation rate of the coating during the period investigated^13^. A previous study confirmed that novel zein–BG porous scaffolds exhibit good bioactivity and suitable mechanical properties^14^. Zein containing Cu-doped 45S5 BG and 45S5 BG fillers showed homogeneous and nearly crack-free surfaces, with BG particles evenly distributed throughout the film. The results showed that hydroxyapatite formed in all composite coatings after seven days of implantation in simulated body fluid (SBF)^1,15^. Therefore, This is almost the first study that focuses on preparation n of BG/zein composite in the form of microspheres “Beads”. The objective of this study is to synthesis a new design of bio-composite zein/SA composite beads loaded with different concentrations (10,20,30, and 40%) of (BG) by dropwise method. Calcium chloride (CaCl_2_, 5%), was used as a crosslinking agent and wight ratio of zein/SA is (1:1) wt. Bioactivity will be studied by immersion of these samples in the Stimulated Body Fluid (in-vitro test). Samples will be characterized by FTIR, XRD, SEM, and EDXA. The interaction forces between zein and sodium alginate (SA) were explored in silico through molecular docking. Molecular dynamics (MD) simulations on beads are used to simulate the mechanical properties of these composites^16–18^.

## Materials and methods

Tetraethyl orthosilicate (TEOS: C_8_H_20_O_4_Si, Mw = 208.33 g/mol), calcium nitrate tetrahydrate (Ca (NO_3_)_2_·4H_2_O, Mw = 236.14892 g/mol), triethyl phosphate (TEP: C_6_H_15_O_4_P, Mw = 182.15 g/mol), and 2 M nitric acid (HNO_3_) were acquired from Merck Inc. (Darmstadt, Germany). Sodium alginate (SA: NaAlg), zein protein, and calcium chloride (CaCl_2_) were obtained from Acros Organics Ltd. (New Jersey, USA). All other chemicals for the preparation of SBF and phosphate-buffered saline (PBS) were purchased from Sigma-Aldrich (St. Louis, MO, USA).

### Synthesis of bioactive glass (60 S-BG)

A bioactive glass was prepared by sol-gel method with chemical composition of (60% SiO_2_ – 35% CaO – 5% P_2_O_5_) wt. according to^19^. Briefly, (22.554) ml of tetra-ethyl-orthosilicate (TEOS, Mw = 208.33 g/mol) was added into (3.897) ml 2 M of nitric acid (HNO_3_, Sigma Aldrich Co USA), 10 ml of ethanol and (23.385) ml of distilled water. The mixture was kept under stirring for 45 min to promote the acid hydrolysis of TEOS. The following reagents were added in a sequence to the above mixture, allowing 45 min for each one to react and completely hydrolysis: (1.264) ml of Triethyl phosphate (TEP C_6_ H_15_ O_4_P, Mw = 182.15 g/mol) and 15.041 g of calcium nitrate tetrahydrate (Ca (NO_3_)_2_. 4 H_2_O) Mw = 236.15 g/mol) as listed in Table 1.

**Table 1:** Synthesis of 60 S BG with sol-gel method.

Reagent source	TEOS	TEP	Ca (NO_3_)_2_. 4 H_2_O	HNO_3_	Distilled water
60 S BG	22.554 ml	1.264 ml	15.041 g	3.897 ml	23.385 ml

**Fig. 1:**
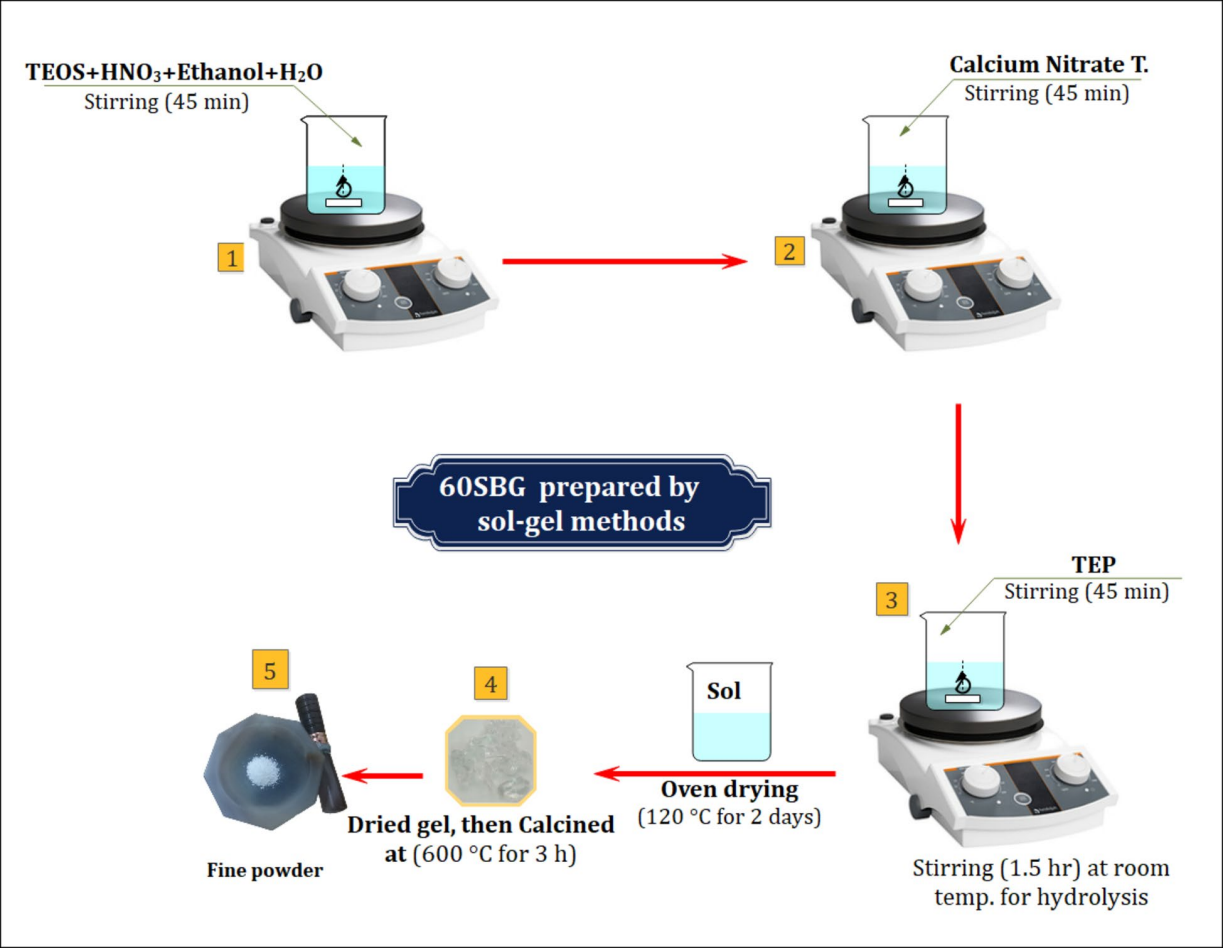
Flow chart of the manufacturing processes for bioactive glass (60 S-BG).

Then the above mixture was kept under stirring for 45 min. The obtained solution was heated at 120 C for 3 days to evaporate the water and organic matter. After that, powder was calcined for 3 h at 600 ℃ to remove the toxic nitrate ions^20^. BG powder was deagglomerated in a motor agate, and sieved at 90 micrometers. The preparation steps are illustrated in the flow chart in Fig. 1.

### Synthesis of BG-loaded Zein/SA composite beads

**Fig. 2:**
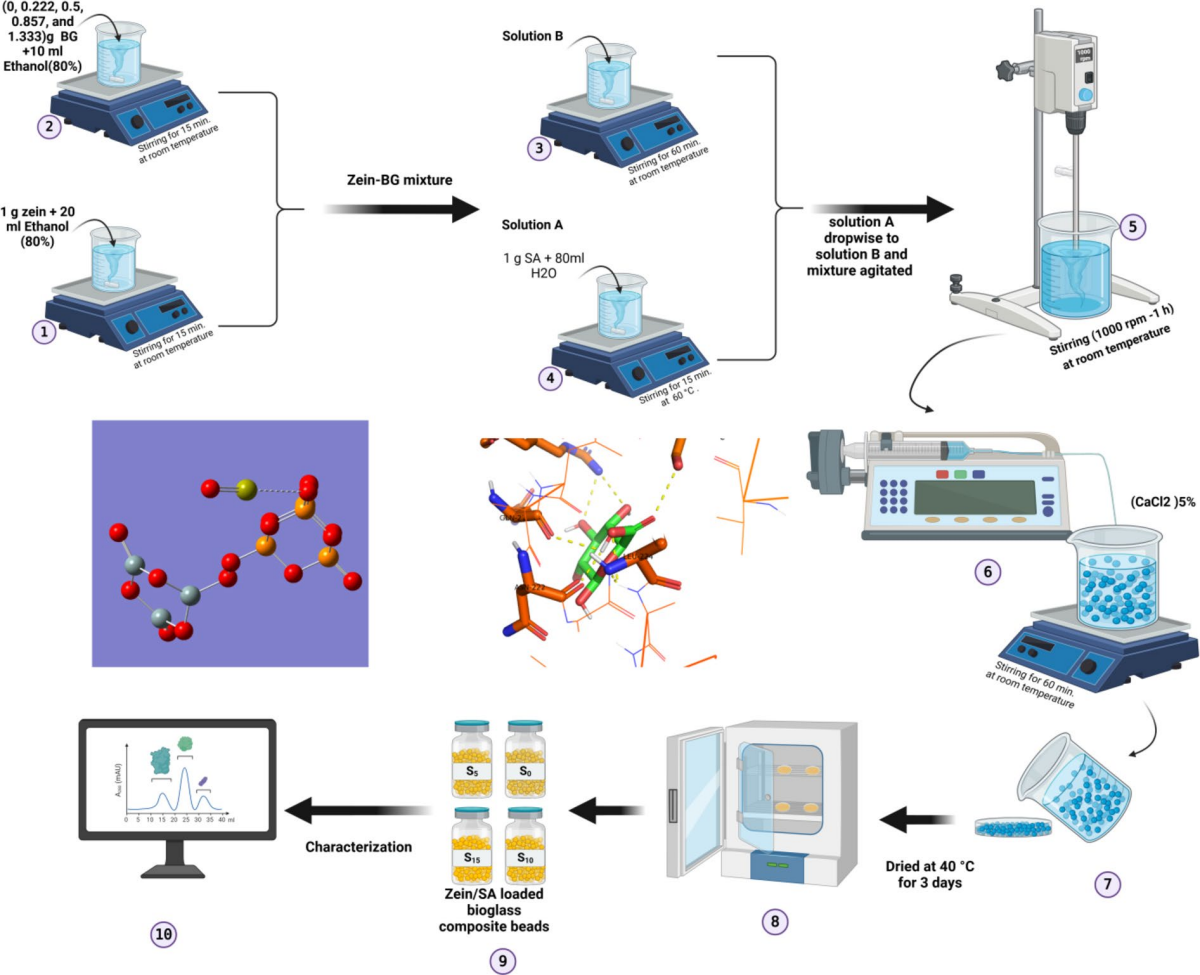
Flow chart of the manufacturing processes for BG-loaded Zein/SA composite beads. Created in BioRender. Desoky, A. (2025) https://BioRender.com/u52z552.

Solution A: 1 g of sodium alginate (SA) was dissolved into 80 ml of distilled water and kept under stirring at 60 C. Solution B consisted of a mixture of 1 g of zein protein which was dissolved into 20 ml of ethanol-water (80%, (v/v)), then added to a solution of bioglass with different concentration Table 2 which were dissolved into 10 ml of ethanol-water (80%(v/v)). Zein- BG mixture (solution B) was kept under stirring for 15 minutes, then added as dropwise gradually to “Solution A” (Fig. 2) (sodium alginate (SA)). the final mixture was mechanically mixed using a homogenizer (High- Torque Digital Overhead Stirrer,” HT-50DX” homogenizer (Germany)) with a rotating speed of 1000 rpm for 1 h until the final mixture had completely homogenized. Calcium chloride (CaCl_2_, 5%), was used as a crosslinking agent^21^. Zein-SA loaded BG mixture was dropped by a syringe pump unit (50 ml/h) into a 125 ml of calcium chloride solution to produce Zein-SA loaded BG beads, and stirred with 140 rpm on a magnetic stirrer at room temp for 2 h^22^. Finally, the obtained BG-loaded Zein/SA composite beads were removed from the solution washed several times with distilled water, and left to dry in a petri dish for 3 days at 40 C and then collected.

### In vitro bioactivity study

**Table 2:** Chemical composition of BG-loaded Zein/SA composite beads in (wt%).

Sample name		Zein/ SA w/w %	BG (g)
S0	0%BG-Zein/SA	1:1	0
S10	10%BG-Zein/SA	1:1	0.222
S20	20%BG-Zein/SA	1:1	0.500
S30	30%BG-Zein/SA	1:1	0.857
S40	40%BG-Zein/SA	1:1	1.333

The prepared samples of BG-loaded zein/SA composite beads were evaluated in vitro by immersing them in simulated body fluid (SBF) following the standard protocol established by^23^. The samples, with a concentration of 2.5 g/L, were placed in sealed containers and submerged in SBF for 33 days at 37 °C. After removal from the SBF, the samples were rinsed with distilled water and allowed to dry.

### Characterization

The identification of the phases, whether amorphous or crystalline, on the surfaces of the BG-loaded zein/SA composite beads was performed using an X-ray powder diffractometer (XRD), specifically the BRUKER D8 ADVANCE model from Germany, equipped with a copper target (Cu Kα = 1.54060 Å) and a nickel filter. The diffraction was measured over a 2θ range from 0° to 60°, with a step size of 0.014° and a time of 1 s per step. For FTIR measurements, the samples were ground, mixed with spectroscopy-grade KBr (Merck, Germany), and pressed into pellets consisting of 1 mg of sample and 200 mg of KBr. Spectra were recorded in absorbance mode over the range of 4000 to 400 cm⁻¹ with a resolution of 4 cm⁻¹. The surface morphology was examined using Field Emission Gun Scanning Electron Microscopy (FEG-SEM) (XL30, Philips) at an accelerating voltage of 30 kV. The specimens were mounted on a stub with a carbon sticker for observation under the microscope. Chemical analysis was conducted using energy dispersive X-ray analysis (EDXA) coupled with the SEM instrument, utilizing a 30 mm² Si (Li) R-RSUTW detector at 15 kV acceleration voltage. EDXA was employed to compare the intensities of calcium (Ca) and phosphorus (P) in the beads before and after invitro test.

#### Protein adsorption

To investigate the physiological behavior of the BG-loaded zein/SA composite beads, protein adsorption on these surfaces was examined. Bovine serum albumin (BSA) was selected as the model protein. A solution containing 0.2 g of BSA in 200 ml of PBS at pH 7.4 and 37˚C was prepared. Then, 0.4 g of each sample was added to 40 ml of this BSA-PBS mixture. The adsorption process was carried out in an incubator at 37˚C for 1 h. Afterward, the samples were washed three times with PBS and once with distilled water to remove any unbound proteins and residual salts and then dried at 37˚C. The amount of protein adsorbed on the sample surfaces was determined using FT-IR spectroscopy.

#### Theoretical 3D modeling and validation of Zein

Alpha zein protein (265 amino acids) was obtained from the NCBI (National Center for Biotechnology Information) in FASTA format. Due to the absence of a crystal structure for zein or highly similar homologous proteins in the protein structure database, the I-TASSER server was employed to predict the protein structure in this study automatically. I-TASSER (Iterative Threading ASSEmbly Refinement) is a method used to predict protein structure and function^24^. It begins by identifying structural templates from the PDB database using multiple threading techniques, then constructs a full-length protein model from fragment simulations based on iterative templates. The protein function database BioLiP was then utilized to further optimize the 3D model of the protein. I-TASSER, a tool developed by the Zhang Lab, has consistently been the top server for protein structure prediction in the Critical Assessment of Structure Prediction (CASP). The best model was selected based on the C-score (confidence score) and refined using the ModRefiner server. Further validation was conducted using the PROCHECK and ERRAT servers.

#### Molecular docking of Zein and SA

To explore the potential driving forces between zein and SA, a docking study was conducted using the Autodock Vina software^25^. SA was downloaded from PubChem (https://pubchem.ncbi.nlm.nih.gov) in SDF format, converted to a 3D PDB format using PyMOL, and used as a ligand. The docked complex was optimized to fit the receptor pocket. PyMOL (The PyMOL Molecular Graphics System, Version 1.8 Schrödinger, LLC) was used to analyze and plot the binding sites and interaction forces. The two-dimensional (2D) and three-dimensional (3D) structures were saved.

## Results and discussion

### FTIR of the (60 S-BG)

**Fig. 3:**
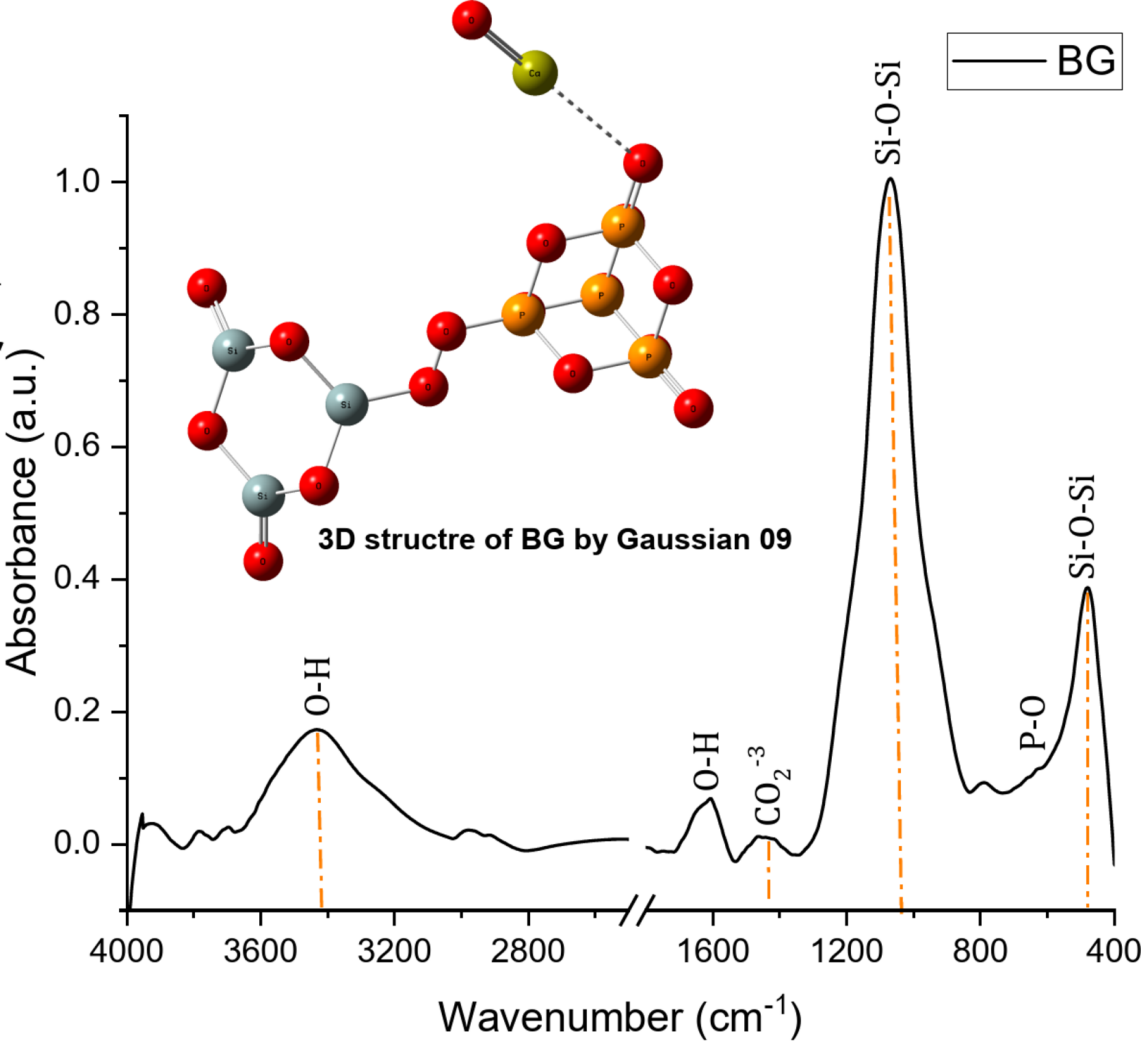
(FTIR) spectrum and 3D structure (Gaussian 09) of 60 S-BG powder (60% SiO_2_, 35% CaO, and 5% P_2_O_5_) prepared by sol-gel method.

FTIR spectrum of the 60 S-BG powder (composed of 60% SiO₂, 35% CaO, and 5% P₂O₅) is depicted in Fig. 3. The broad band ranging from 3100 cm⁻¹ to 3600 cm⁻¹ can be attributed to O–H stretching^19,26^, which is associated with strong hydrogen bonds of both intramolecular and intermolecular types. The band at 1648 cm⁻¹ is linked to O–H bending vibrations of hydroxyl groups that are chemically adsorbed onto the glass matrix^27^. The peak observed at 1400–1530 cm⁻¹ is associated with carbonate (CO₃)²⁻ groups. The presence of carbonate is attributed to the carbonation of the material caused by atmospheric CO₂, resulting from its high calcium content^28,29^.

Absorption bands of 60 S-BG were detected around 1000–1100 cm⁻¹, 787 cm⁻¹, and 478 cm⁻¹, corresponding to Si–O–Si and Si–O-Ca stretching modes, as well as the Si–O–Si bending mode, respectively^26,30^. These bands are commonly found in amorphous silica glasses^31,32^. The δ(Si-O-Si) band appears around 800 cm⁻¹, indicating the formation of a highly disordered three-dimensional silica structure^33^.

### FTIR of the composite beads

**Fig. 4:**
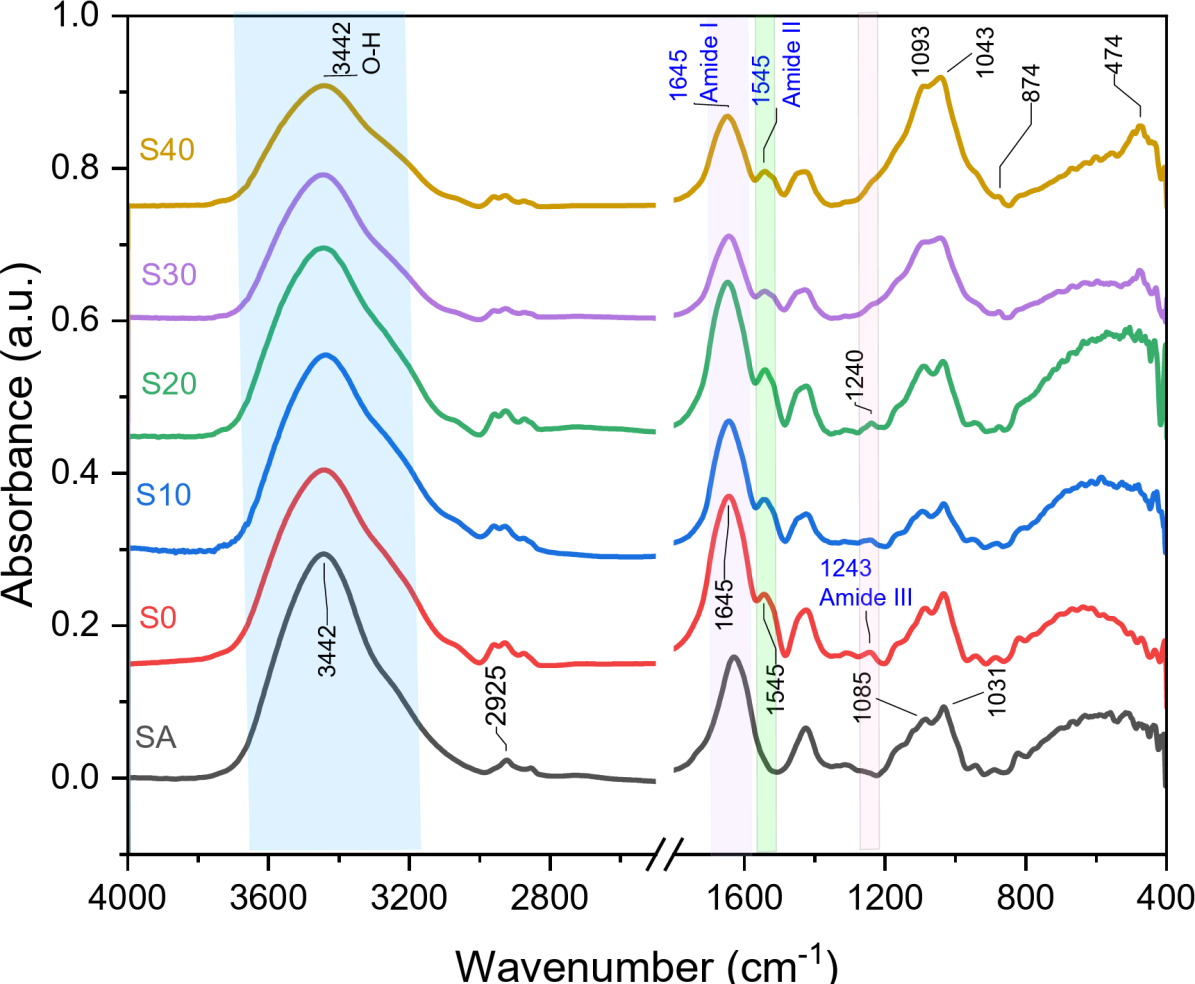
(FTIR) patterns of (SA), S0, S10, S20, S30, and S40 composite beads before immersion in SBF.

Figure 4 shows the FTIR spectra for sodium alginate (SA), S0, S10, S20, S30, and S40 composite beads. Silicate absorption bands were detected at 474, 875, and 1093 cm^− 1^, corresponding to the asymmetric stretching mode, symmetric stretching vibration, and rocking vibration of Si-O-Si, respectively^34^. A weak, sharp band at 556 cm^− 1^ is attributed to the P-O bending vibration^35^. Additionally, an absorption band in the 3000–4000 cm^− 1^ range indicated the stretching vibration of the O-H group^13^. The intensity of these absorption bands increased with higher BG concentrations. The primary characteristic bands of sodium alginate (SA) appeared at approximately 3442, 2925, 1085, and 1031 cm^− 1^. The band at 3442 cm^− 1^ is associated with the stretching vibration of the hydroxyl group (OH)^36–38^, while a weaker band at 2925 cm^− 1^ indicates the stretching vibrations of C-H^39^. Two strong bands at 1085 and 1031 cm^− 1^ correspond to the stretching vibrations of C-O, C-C, and COC^40,41^. It was observed that all major SA bands gradually decreased with the addition of BG. The significant bands of zein appeared in the FTIR spectra at about 1645 cm^− 1^ (C = O stretch)^13^, 1545 cm^− 1^ (N-H bend and C-N stretch)^14,42^, and 1243 cm^− 1^ (N-H bend and C-N stretch)^43^, corresponding to amide I, amide II, and amide III, respectively.

#### Deconvolution

In the amide I region, the components identified within the ranges of 1640–1620 cm^− 1^ and 1700–1693 cm^− 1^ are associated with β-sheet structures, those between 1660 and 1650 cm^− 1^ are linked to the α-helix structure, and the range from 1690 to 1660 cm^− 1^ corresponds to the β-turn structure^44–47^.

**Fig. 5:**
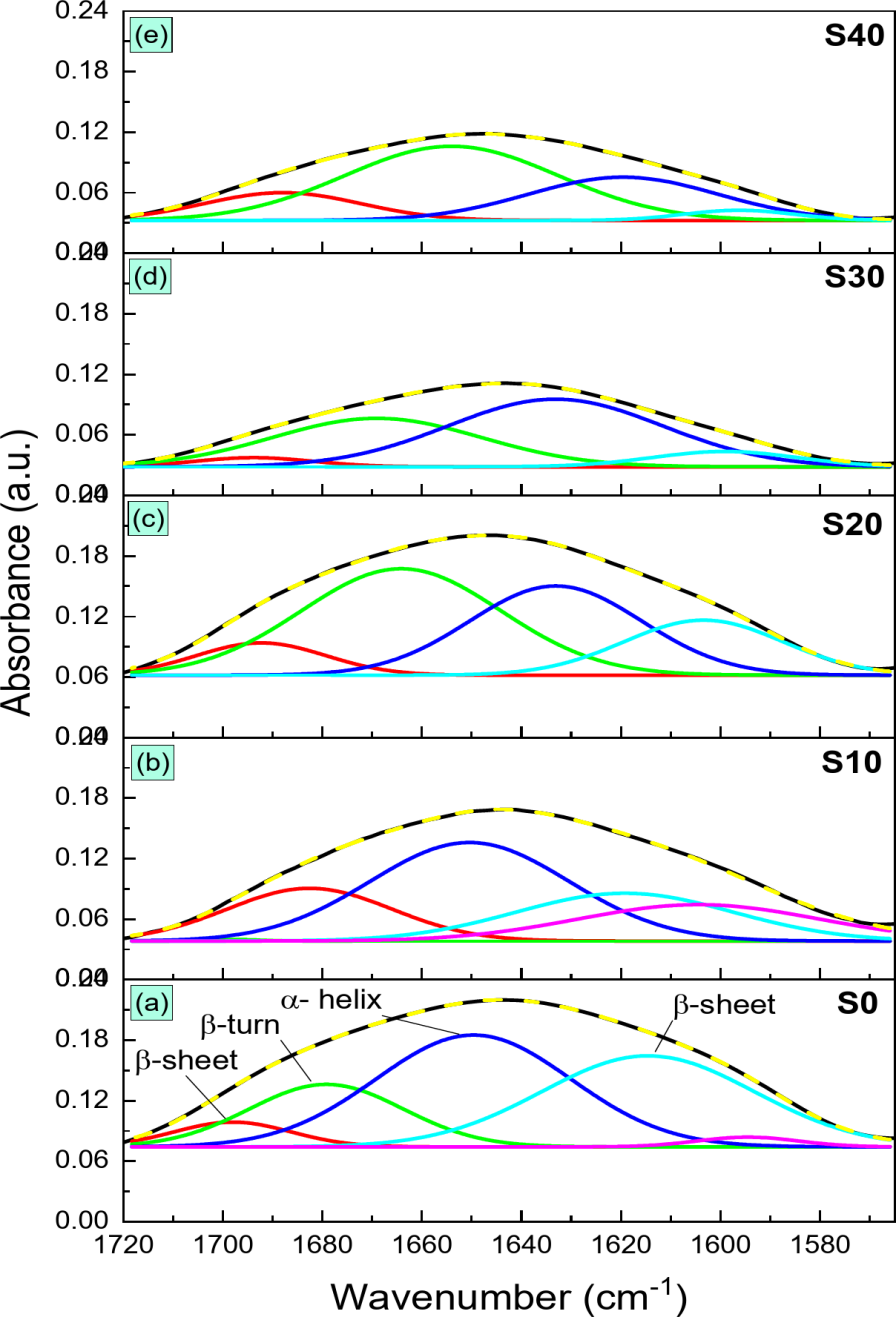
Amide I band region deconvolution of zein protein in (a) S0, (b) S10, (c) S20, (d) S30, and (e) S40, composite beads.

Bands observed at 1698, 1679, 1650, and 1614 cm^− 1^ in the deconvoluted amide I spectrum of zein protein indicate β-sheet, β-turn, α-helix, and β-sheet structures, respectively (see Fig.5). A noticeable shift in the α-helix band from 1650 cm^− 1^ to 1619 cm^− 1^ suggests an interaction between the zein protein and BG.

**Fig. 6:**
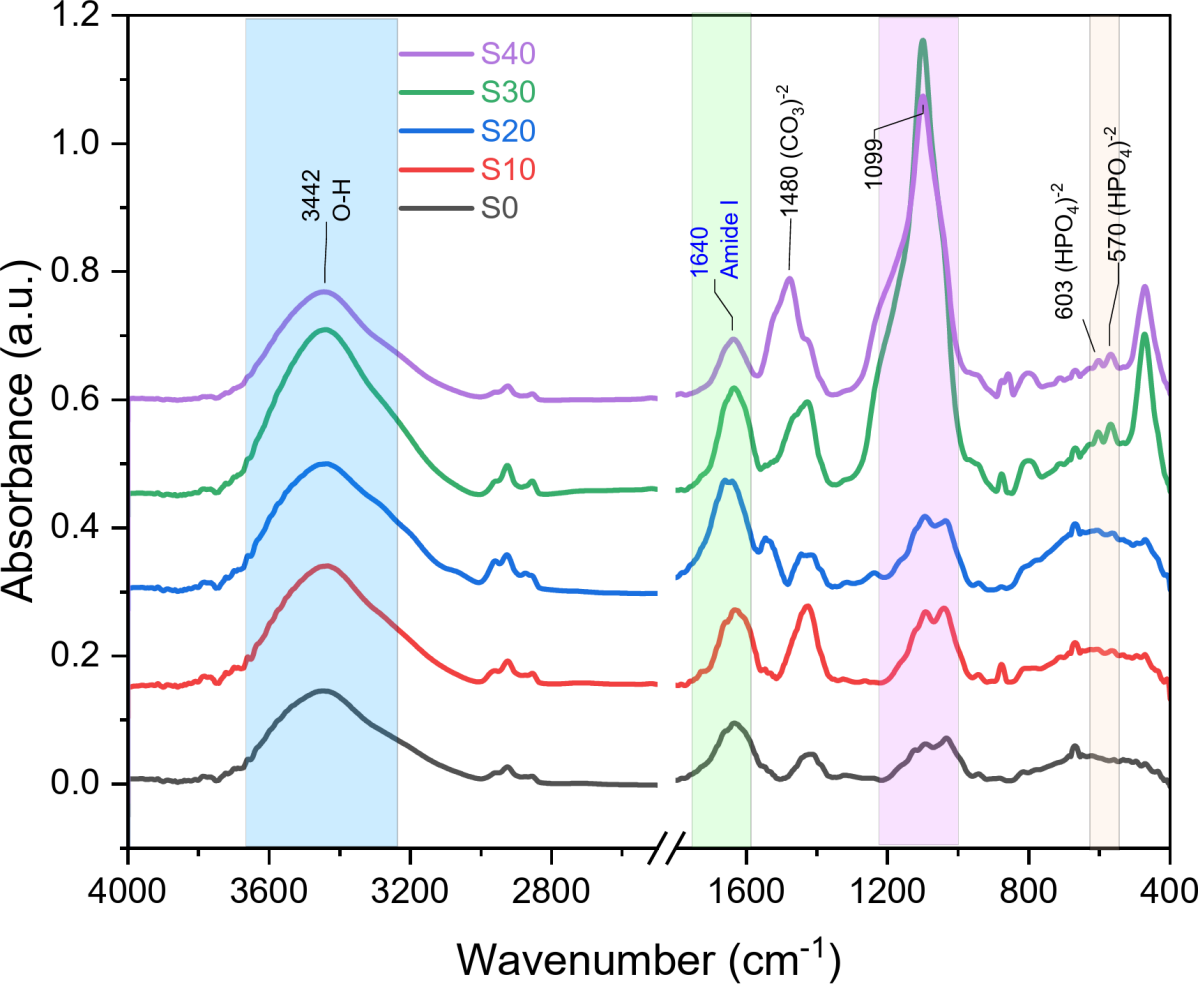
(FTIR) spectra of S0, S10, S20, S30, and S40 composite beads after immersion in SBF.

The FTIR spectra of zein/SA and zein/SA loaded with BG composite beads after 33 days of immersion in SBF are shown in Fig. 6. Several characteristic bands of SA and zein either decreased or disappeared. For instance, significant SA bands at 1031 and 1085 cm^− 1^ gradually diminished and eventually disappeared in the zein/SA BG 40% beads. A new SA characteristic band appeared at 1633 cm^− 1^, overlapping with a zein protein band at 1645 cm^− 1^ before immersion in SBF. The zein protein bands at 1243, 1545, and 1645 cm^− 1^ disappeared, indicating degradation of the zein protein during immersion in SBF. New significant bands were observed at 570 and 603 cm^− 1^ for P-O, indicating the presence of (δ P-O crystal)^20^, which supports the formation of HAP^23^.

### Protein adsorption

**Fig. 7:**
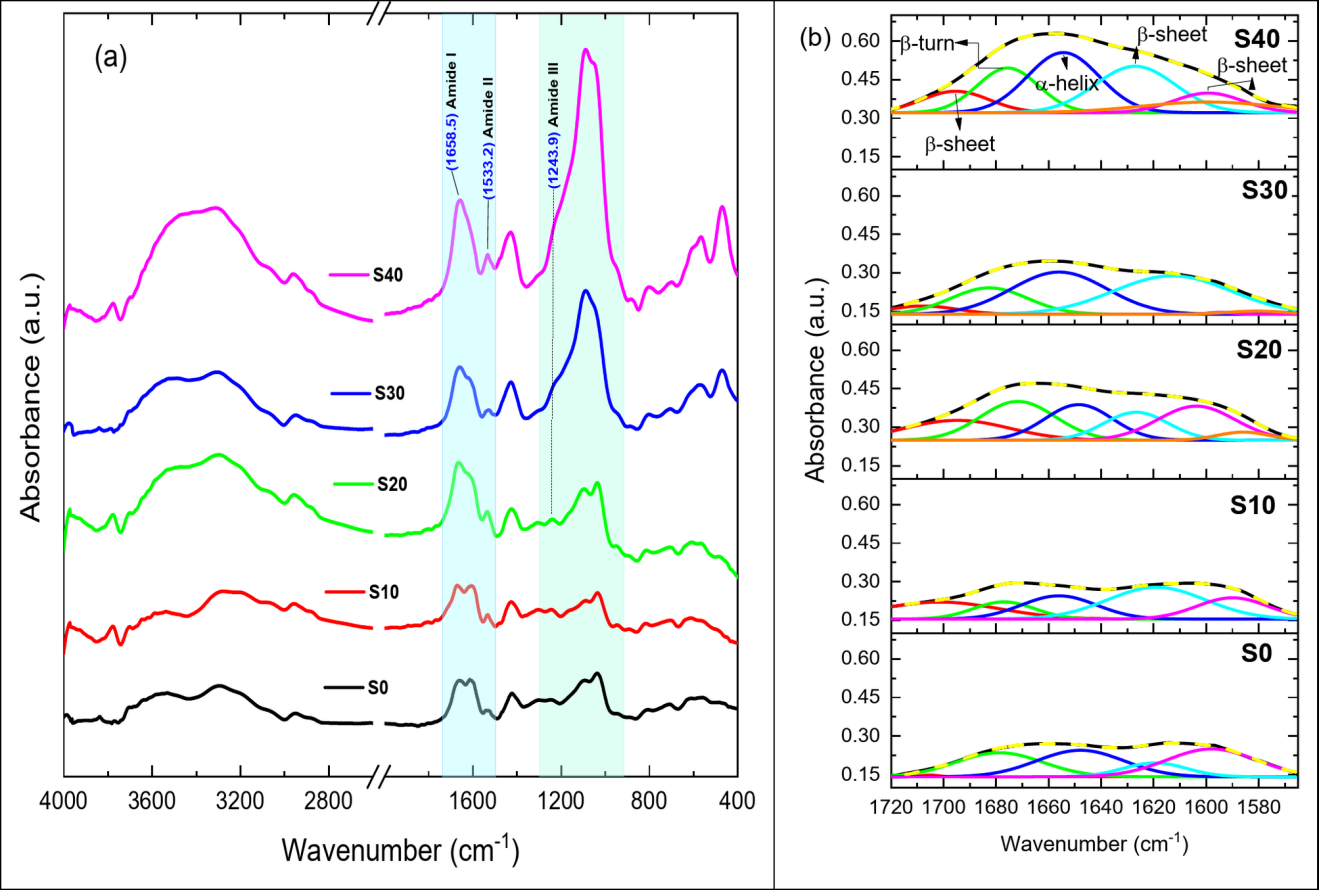
FTIR spectra of Adsorption for BSA adsorption onto S0, S10, S20, S30, and S40 composite beads (a) Amide I band region deconvolution (b).

Significant changes in the FTIR spectra of Bovine serum albumin (BSA) adsorption onto S0, S10, S20, S30, and S40 composite beads were observed in the 1700 to 1600 cm^− 1^ range (Fig. 7a). As the BG content increased, the intensity of the amide I and amide II bands also increased. The amide III band became more pronounced until it formed a weak shoulder at higher BG concentrations (30% and 40%), overlapping with the silicate band. Deconvolution of the amide I band region (Fig. 7b) revealed that the intensity of the α-helix band gradually increased with BG content, possibly due to BSA adsorption. In physiological conditions, BGs usually have a negative surface charge^48^. However, the surface charge of proteins is more complex because the uneven charge distribution within protein molecules creates differently charged domains on their surfaces.

Zein consists mainly of α-zein, which has an isoelectric point at neutral pH^49^, where carboxyl groups are partially ionized, giving alginate its characteristic negative charge^50^. Additionally, micropores on the surfaces of BG-zein/SA beads at 10%, 20%, 30%, and 40% BG content enhance overall porosity, as confirmed by SEM analysis. Increased porosity boosts protein adsorption, mainly due to a subsequent increase in specific surface area (SSA), and also affects the structure of the adsorbed proteins. Finally, the permeability of BSA adsorption onto the surface of BG-zein/SA beads at 10%, 20%, 30%, and 40% of BG content is related to variations in BG content, which have a greater effect than the zein protein and sodium alginate.

### XRD of the composite beads

**Fig. 8:**
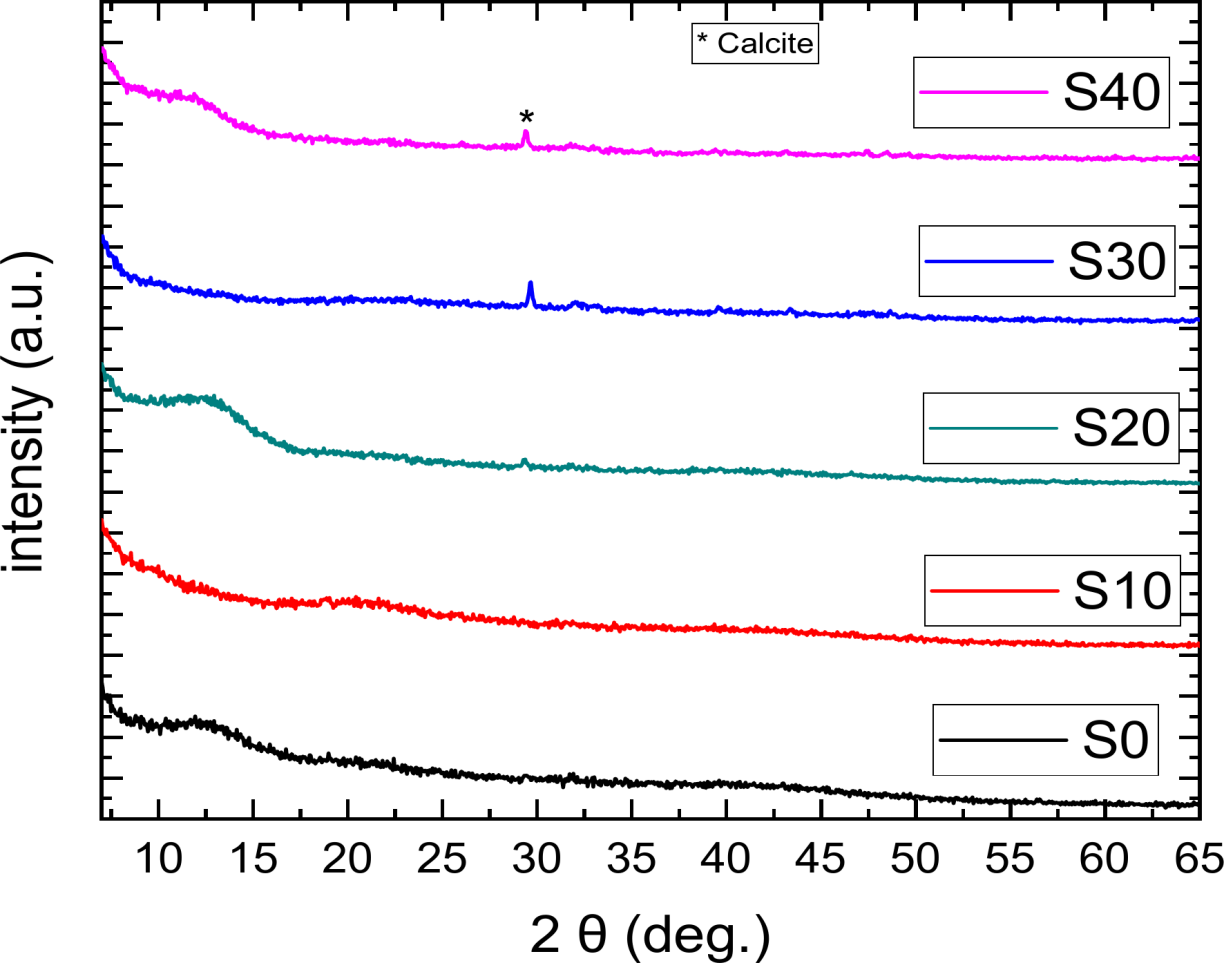
(XRD) patterns of S0, S10, S20, S30, and S40 composite beads before immersion in SBF.

The Fig. 8 displays the XRD patterns of S0, S10, S20, S30, and S40 composite beads. Before immersion, the XRD spectra for all samples showed no sharp diffraction peaks, indicating that the BG particles have a typical amorphous structure.

A broad peak appeared at around 2θ of 23°^51^, which is characteristic of the amorphous structure of silicate glass. Additionally, a broad peak in the 2θ range of 8°-20° was observed, indicating the crystalline nature of the protein structure^52^.A weak band was observed at around 29° related to calcite^53^.

**Fig. 9:**
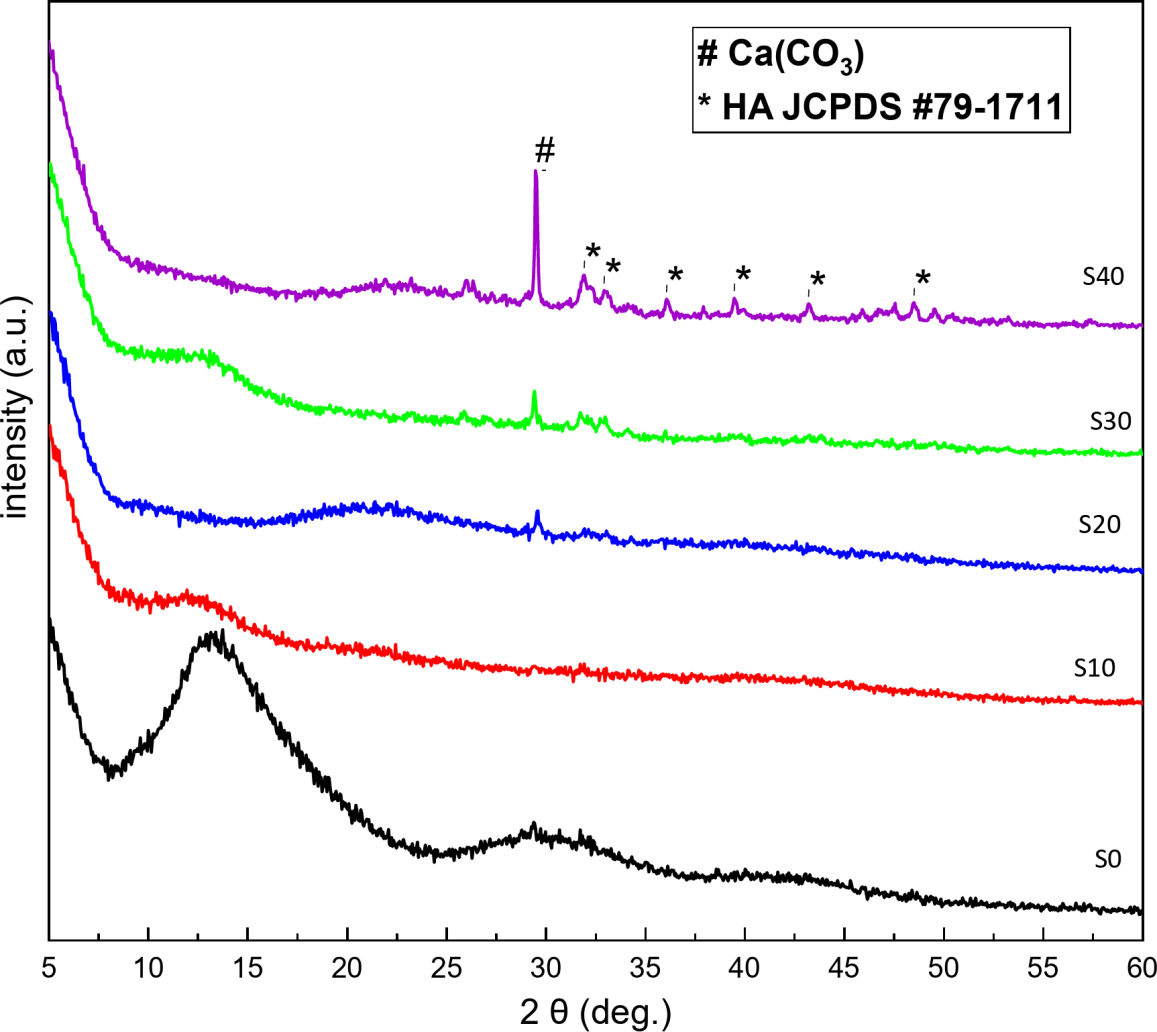
(XRD) patterns of S0, S10, S20, S30, and S40 composite beads after immersion in SBF.

The Figure 9 shows the XRD patterns of S0, S10, S20, S30, and S40 composite beads after 33 days of immersion in SBF. Weak characteristic peaks of HAP were observed at 2θ values of 25.9°, 32.2°, 33°, 38°, 39.4°, 43.2°, and 48.4°, consistent with JCPDS (09-0432). The intensity of these HAP peaks increased gradually from S10 up to S40 composite beads as the BG content rose. Additionally, a new sharp peak at 29° 2θ likely corresponds to calcium carbonate (CaCO_3_), formed from the interaction between the calcium in BG and the carbonate in the zein protein. The reduction in the intensity of the HAP peaks may be due to decreased degradation of the zein protein in the BG-zein/SA complex structure. Finally, S40 composite beads exhibit higher activity than the other beads due to their higher BG content.

### SEM & EDXA of the composite beads

**Fig. 10:**
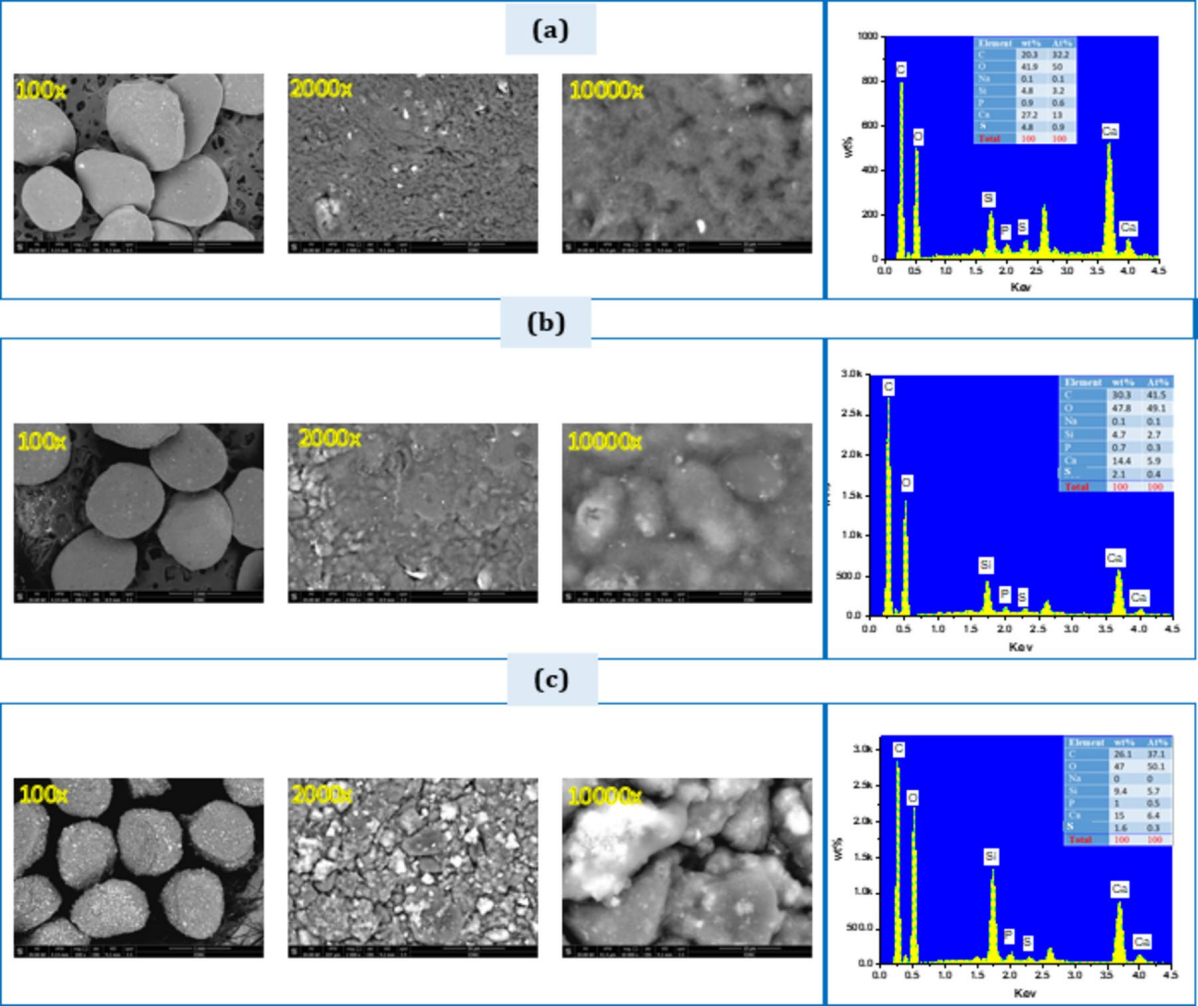
(SEM) images and EDXA profiles of, (a) S10, (b) S20, (c) S40 composite beads before immersion in SBF.

Figure 10 shows the SEM spectra and EDXA of the zein/SA composite beads loaded with varying concentrations of BG (10%, 20%, and 40% by weight). The results indicate that the beads are spherical and uniform in size, with an average diameter of about 900 micrometers. The surface of the BG-zein/SA (S10, S20, and S40) beads contains micropores, which enhance the porosity of all the beads. The presence of SA and zein protein, along with their crosslinking with BG, is expected to promote the formation of these micropores, thereby increasing porosity. EDXA analysis revealed the presence of key elements of BG, such as network silicate (Si), calcium (Ca), and phosphorus (P) ions. The main elements of zein and sodium alginate, like carbon (C), sulfur (S), and sodium (Na) were also detected due to the crosslinking of zein and SA with BG. It was noted that the peaks for silicate (Si), Ca, and P increased as the BG concentration rose.

**Fig. 11:**
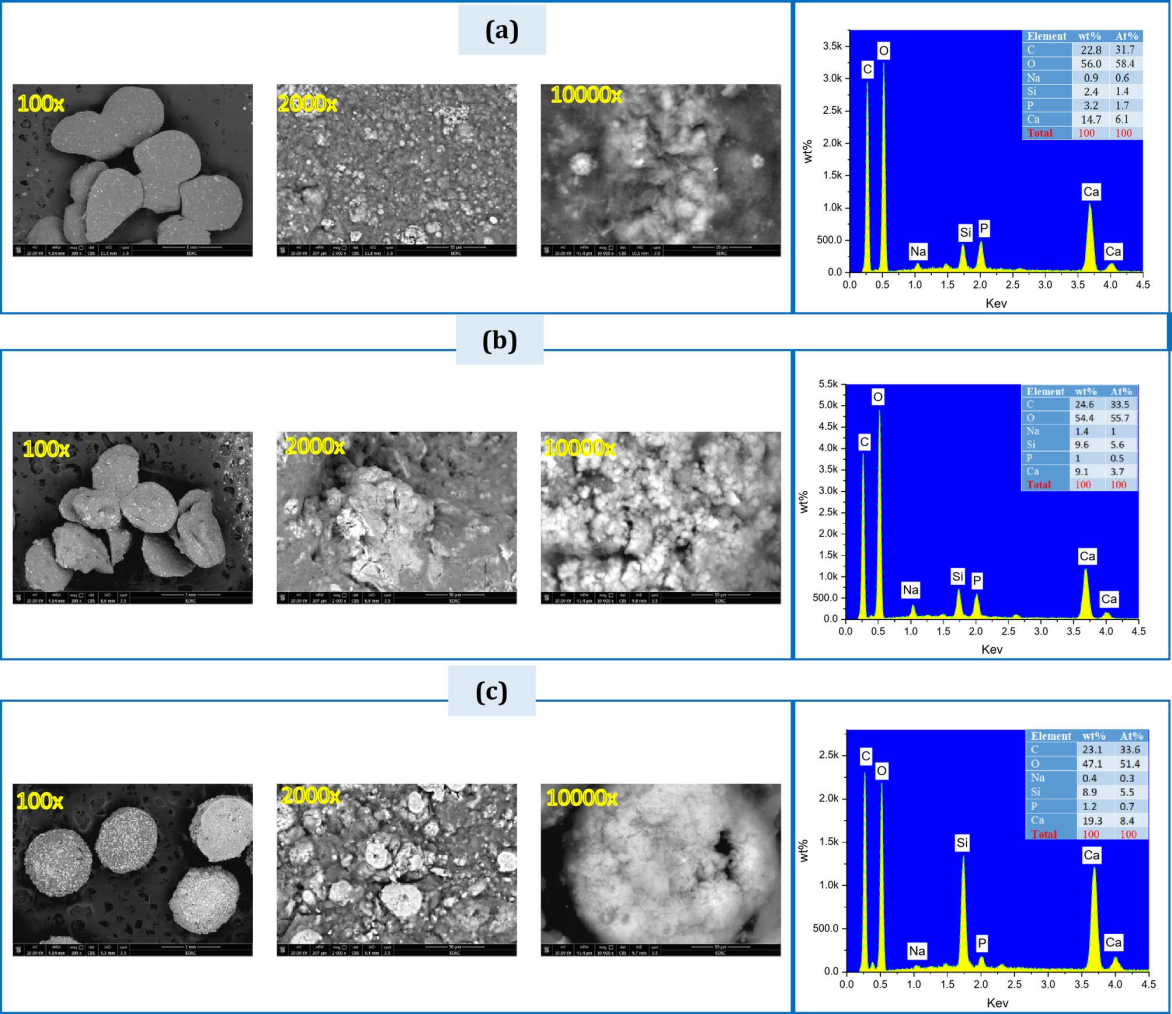
(SEM) images and EDXA profiles of, (a) S10, (b) S20, (c) S40 composite beads after 33 days of immersion in SBF.

Figure 11 demonstrates the SEM spectra and EDXA results after immersing the BG-zein/SA (S10, S20, and S40) beads in SBF for 33 days. The average diameter of the beads decreased to about 500 micrometers, possibly due to the degradation of SA and zein protein. A layer of HAP was deposited on the surface of the BG-zein/SA (S10, S20, and S40) beads in the form of flake-like crystals. This layer completely covered the BG-zein/SA (S40) beads and partially covered the BG-zein/SA (S20) and BG-zein/SA (S10) beads. The EDXA results of the BG-zein/SA (S10, S20, and S40) beads after immersion in SBF showed increased peaks for calcium (Ca) and phosphorus (P), reflecting their role in the HAP deposition process. In vitro testing revealed the formation of a hydroxyapatite (HA) layer on the surface of the composite beads after immersion in Simulated Body Fluid (SBF). This HA formation is a hallmark of bioactivity, indicating the material’s compatibility with biological systems.

### Zein structure prediction, refinement, and validation

**Fig. 12:**
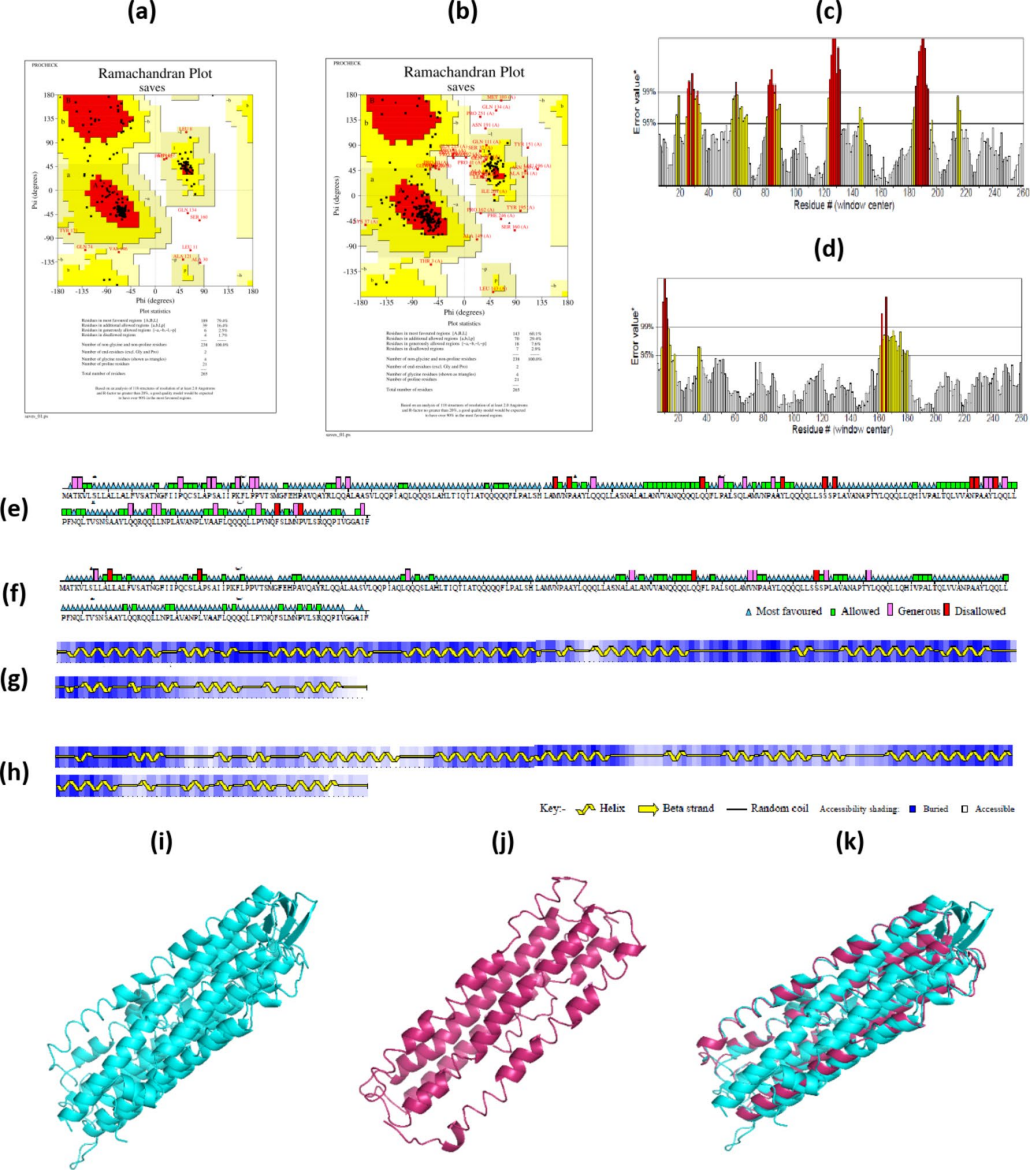
The Ramachandran diagrams of zein structures (a) before and (b) after optimization. Comparisons of amino acid sequence error rates (c) before and (d) after optimization. The proportion of amino acid that fell in the most favored regions, additional allowed regions, generously allowed regions, and disallowed regions (e) before and (f) after optimization. The secondary structures of zein (g) before and (h) after ModRefiner server. (i) illustrates the structure of zein obtained from I-TASSER modeling, and (j) describes the structure after optimization. (k) denotes the overlapping conformations of the two structures.

A BLAST (Basic Local Alignment Search Tool) search against the PDB database showed no significant similarity with zein. Due to the lack of an experimental 3D structure, modeling was performed using the I-TASSER server, resulting in a modeled zein with a C-score of -2.91. The C-score is a confidence score used by I-TASSER to estimate the quality of predicted models, calculated based on the significance of threading template alignments and the convergence parameters of the structure assembly simulations. For refining the theoretical model structure, ModRefiner indicated a RMSD (Root Mean Square Deviation) of 1.376 Å and a TM score of 0.9691 Å from the initial model. The superimposed structures of the theoretical and refined models are shown in Fig. 12.

The Ramachandran diagram was used to analyze the protein backbone structure, and the dihedral angles (φ, Ψ) of amino acid residues in different regions correspond to different structures. The diagram’s colors range from red, dark yellow, and light yellow, to white, representing the most favored, additionally allowed, generously allowed, and disallowed regions, respectively^54^. Figure 12a showed that 60.1% of amino acid residues were in the most favored regions of the unoptimized zein backbone, with an overall quality factor of 78.8. After optimization through ModRefiner server, this percentage increased to 79.4%, with an overall quality factor of 90.5 (Fig. 12b). A score above 90 indicates excellent quality for this protein structure according to a previous study^55^. In the Ramachandran diagram, the upper left part represents the β-strand region, where all amino acid residues form a β-strand structure; the lower left part represents the right-hand α-helix region, and the upper right part indicates the less common left-hand α-helix region^56^. It was observed that the α-helix content was the highest. Additionally, the amino acid sequence error rate in the protein’s primary structure decreased after optimization (Fig. 12c versus d). Furthermore, the proportion of residues in the most favored regions increased, while the proportion in disallowed regions decreased Fig. 12e versus f). Figures 12g and h illustrate the secondary structure contents for various amino acid numbers. Following optimization, the protein backbone became more ordered, resulting in an increase in β-strand content and unordered coils, as shown in Figs. 12a and b. Figures 12i and j depict the tertiary structure before and after optimization. Figure 12k shows the overlapping of protein conformations, allowing for visualization of the structural changes. The optimized structures were subsequently used for docking.

### Molecular docking

**Fig. 13:**
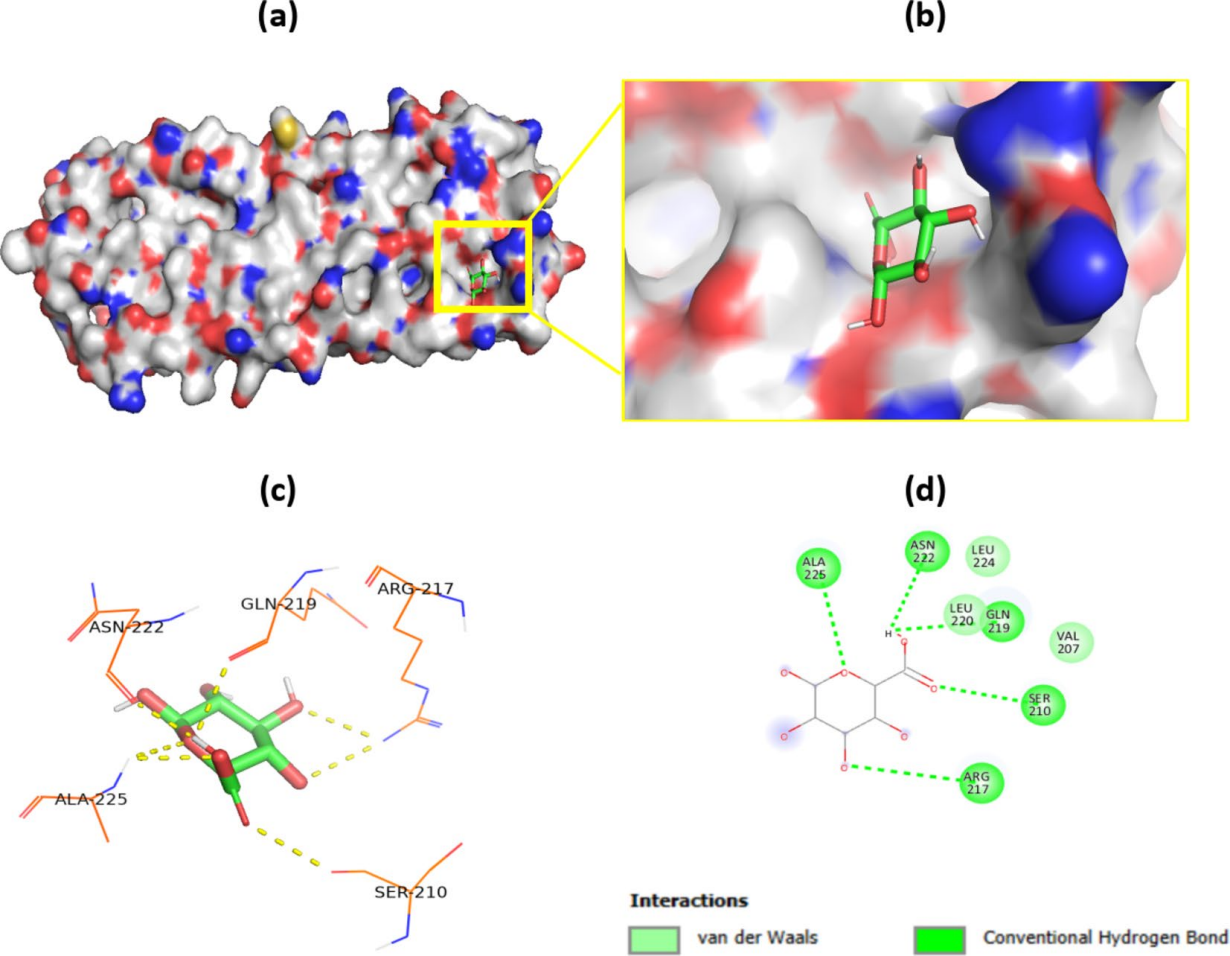
Predicted binding mode of SA to the active site of zein in the 3D model (a and b). Specific interaction sites (c) and forces (d) between SA and zein in a 2D docking model.

A molecular docking study was conducted to gain a detailed understanding of the interaction between SA and zein. As shown in (Fig. 13a, b), the proposed binding pose of SA fits well into the binding pocket of zein. The docking results (Fig. 13c, d) revealed that SA is predicted to form van der Waals interactions with zein at LEU 224, LEU 220, and VAL 207, as well as hydrogen bonds with ALA 225, ASN 222, GLN 219, SER 210, and ARG 217. Overall, the docking results clarified the role of van der Waals forces in the formation of stable complexes. This confirms the presence of the properties of zein (such as degradation), in addition to the properties of sodium alginate (such as mechanical properties).

## Conclusion

New designs of BG-loaded zein/sodium alginate (SA) composite beads were created using a dropwise method. Different concentrations of bioglass (BG) (10%, 20%, 30%, and 40%) were incorporated. Calcium chloride (CaCl_2_, 5%) served as a crosslinking agent, and the weight ratio of zein to SA was maintained at 1:1. Both in-vitro and in-silico analyses were conducted on the BG-loaded zein/SA composite beads. Bioactivity was evaluated by immersing the samples in SBF (in-vitro test). The BG-loaded zein/SA composite beads were characterized using various techniques (XRD, FTIR, SEM, and EDXA) both before and after the in-vitro test. The findings revealed the formation of a hydroxyapatite (HA) layer on the surface of the zein/SA composite beads, confirming their bioactivity, which increased with higher BG content. Therefore, adding bioglass enhances the bioactivity of zein/sodium alginate (SA) composite beads, making them suitable for biomedical applications. FTIR and SEM analyses showed degradation of the zein protein and sodium alginate matrix in SBF, which supports the biodegradability of the beads. BG-loaded zein/sodium alginate (SA) composite beads could be explored in future in vivo studies. In silico study, The ModRefiner server delivered more accurate protein structures for zein, which were initially modeled using I-TASSER. Molecular docking studies showed that sodium alginate (SA) interacts with zein via van der Waals forces and hydrogen bonds, resulting in the formation of zein/sodium alginate (SA) composite beads as a stable complex. In future studies, we will investigate in vivo and conduct the necessary measurements for that. In silico we will study the interaction between BG and zein protein and BG, SA and zein protein. And predict mechanical behavior using molecular dynamic (MD) simulation.

## Data Availability

the data presented in this study are available on request from the corresponding authors.
